# Supplementary material for: Tie-2 regulates endothelial morphological responses to shear stress by FOXO1-triggered autophagy
Source: PLoS One. 2025 May 5;20(5):e0322869. doi: 10.1371/journal.pone.0322869 (PMC12052130; doi:10.1371/journal.pone.0322869)
Supplement: S2 Fig — FOXO1 knock-down efficiency. Immunoblotting for FOXO1 and α-tubulin as a loading control of HUVECs transfected with control or FOXO1 siRNA for 48 hours. Data are representative for three similar experiments. (PDF) [file pone.0322869.s002.pdf]

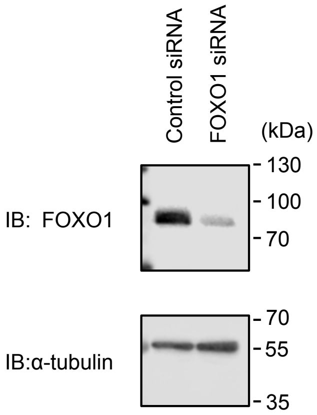

S2 Fig. siRNA-mediated FOXO1 knock-down efficiency Immunoblotting for FOXO1 and α-tubulin as a loading control of HUVECs transfected with control or FOXO1 siRNA for 48 hours. Data is representative of at least three experiments.
