# Supplementary material for: Tie-2 regulates endothelial morphological responses to shear stress by FOXO1-triggered autophagy
Source: PLoS One. 2025 May 5;20(5):e0322869. doi: 10.1371/journal.pone.0322869 (PMC12052130; doi:10.1371/journal.pone.0322869)
Supplement: S3 Fig — Scale bar indicates 50 μm. Data are representative for three similar experiments. (PDF) [file pone.0322869.s003.pdf]

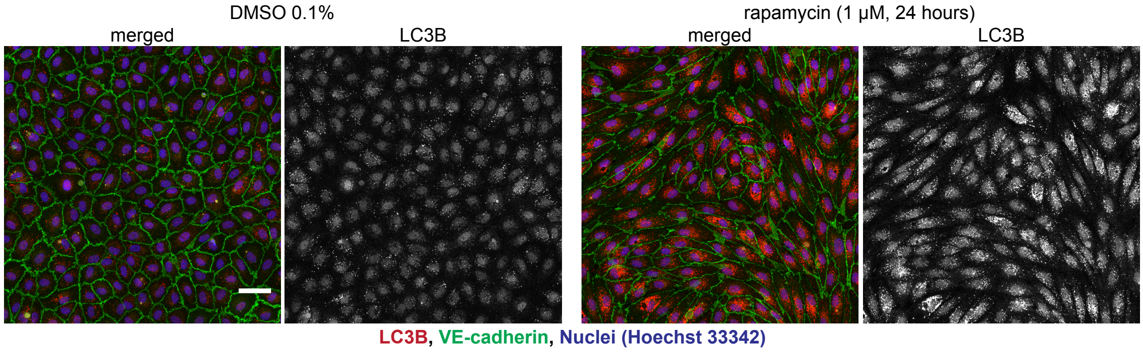

S3 Fig. LC3B antibody validation LC3B immunofluorescence staining of HUVECs treated with rapamycin at 1 µM for 24 hours. Scale bar indicates 50 µm. Data is representative of at least three experiments.
