# Supplementary material for: Tie-2 regulates endothelial morphological responses to shear stress by FOXO1-triggered autophagy
Source: PLoS One. 2025 May 5;20(5):e0322869. doi: 10.1371/journal.pone.0322869 (PMC12052130; doi:10.1371/journal.pone.0322869)
Supplement: Attachment — Submitted filename: Response to reviweres.pdf [file pone.0322869.s006.pdf]

Dr. Saiedeh Razi-Soofiyani
Academic Editor
PLOS ONE

Revised manucript PONE-D-24-43059

“Tie-2 regulates endothelial morphological responses to shear stress by FoxO1-triggered autophagy’

Response to reviewers

We thank the editor and referees for their comments to our manuscript. We have addressed the comments raised by the editor as given below.

Comment 1. Abstract: please rewrite the abstract section based on following format: Introduction, Methods, Results and conclusion. You should not your previous findings in this part.

Response: We modified the abstract according to your comments.

Comment 2. Please use Mesh terms as keywords and put them based on alphabetic order.

Response: We now added MeSH terms as keywords:

Blood vessel (A07.015), Autophagy (G04.011), Cell shape (G04. 320), Signal Transduction (G04.835) and Mechanotransduction, Cellular (G04.835.580).

Comment 3. Introduction section is boring, please summarize this part in 1 page.

Response: We revised the introduction section accordingly.

Comment 4. In Methods section, please indicate the complete information of the software which used.

Response: We added the information about the versions of all software we used in this study.

Comment 5. Try to put . at the end of sentences.

Response: We did this now.

Comment 6. Compare your results with more slimier studies, what is difference or similarity?

Response: We have now re-written parts of the discussion and descibed in more detail what is novel and how this fits to published results.

Comment 7. Use updated references, Please replace out of date references with updated ones.

Response: We revised the references accoringg to the style of PLOS ONE.

Comment 8. What is control protein for western blotting?

Response: In this study, we used alpha-tubulin as a control proten for western blotting. To clarify this, we revised the figure legends.
